# ATR-FTIR spectral discrimination between normal and tumorous mouse models of lymphoma and melanoma from serum samples

**DOI:** 10.1038/s41598-017-17027-4

**Published:** 2017-12-05

**Authors:** Hemendra Ghimire, Mahathi Venkataramani, Zhen Bian, Yuan Liu, A. G. Unil Perera

**Affiliations:** 1 Department of Physics and Astronomy, GSU, Atlanta, GA 30303 USA; 2 Center of Diagnostics and Therapeutics, Georgia State University, Atlanta, GA 30302 USA; 3 Center for Inflammation, Immunity and Infection, Georgia State University, Atlanta, GA 30303 USA

## Abstract

This study presents, attenuated total reflection Fourier transforms infrared spectroscopy of dried serum samples in an effort to assess biochemical changes induced by non-Hodgkin’s lymphoma and subcutaneous melanoma. An EL4 mouse model of non-Hodgkin lymphoma and a B16 mouse model of subcutaneous melanoma are used to extract a snapshot of tumor-associated alteration in the serum. The study of both cancer-bearing mouse models in wild types and their corresponding control types, emphasizes the diagnostic potential of this approach as a screening technique for non-Hodgkin lymphoma and melanoma skin cancer. Infrared absorbance values of the different spectral bands, hierarchical clustering and integral values of the component bands by curve fitting, show statistically significant differences (student’s t-test, two-tailed unequal variance p-value < 0.05) between spectra representing healthy and tumorous mouse. This technique may thus be useful for having individualized route maps for rapid evaluation of lymphoma and melanoma status and associated therapeutic modalities.

## Introduction

The incidence rates of cutaneous melanoma^1^, a deadly form of skin cancer, has been increasing in many regions and populations over the last few decades^2^. The increase has been of the order of 3–7% per year among fair-skinned populations^3^. At the same time, non-Hodgkin’s lymphoma (NHL)^4^, a solid tumorous condition of the immune system with a wide range of histological appearance and clinical features, accounts 4.3% of all new cancer cases in the US^5^. Although significant improvement has been made to stabilize the number of NHL cases and to increase its five-year survival rate, the existing diagnostic techniques, which include the histological examination using biopsy, are time-consuming, invasive, costly, and are not accessible to the entire at-risk population. Developing a rapid and reliable prescreening strategy for melanoma and lymphoma is thus critical because of early diagnosis and treatment of these malignancies better improve^6,7^ the patient’s chances of survival.

Fourier Transform Infrared (FTIR) spectroscopy is an attractive technique for a rapid, reliable and affordable screening of multiple diseases^8–11^. This technique extracts a snapshot of molecular components within the diagnostic medium and provides a holistic biochemistry of that medium^12^. The FTIR spectroscopy combined with appropriate data handling frameworks has been widely applied in many oncological studies^9^ such as studies involving the cancers of the cervix^13^, the lung^14^, the breast^15^, the skin^16^, the gastro-intestine^17^, the prostate^18^, the colon^19^, the ovary^20^, the urinary bladder^21^ and many other body parts. These studies have reported that the molecular structural rearrangement associated with cancer development alters the vibrational mode of the molecular functional groups of the affected tissues as manifested in spectral markers or signatures. Furthermore, the Attenuated Total Reflection (ATR) sampling mode^22^ of FTIR spectroscopy represents a complementary approach for the clinical application^10,23^, compared to other infrared approaches^24^. In this mode, high-quality results with better spectral reproducibility compared to other modes can be obtained by the use of fluid samples^25^. It has been noted that metabolic discharges into the body fluids (saliva, excreta, blood and other tissue fluids) from the proximate cancerous tissue change the constituent molecules, providing strong guidance for subsequent clinical assessment^21,26^. ATR-FTIR spectroscopy of body fluids has thus attracted much attention in the scientific community including clinicians for rapid detection of various health conditions^27^. Herein, we demonstrate the diagnostic capability of ATR-FTIR spectroscopy for the melanoma and NHL by testing air-dried serum samples from respective mouse models.

## Results

### Discrimination of absorbance values

**Figure 1:**
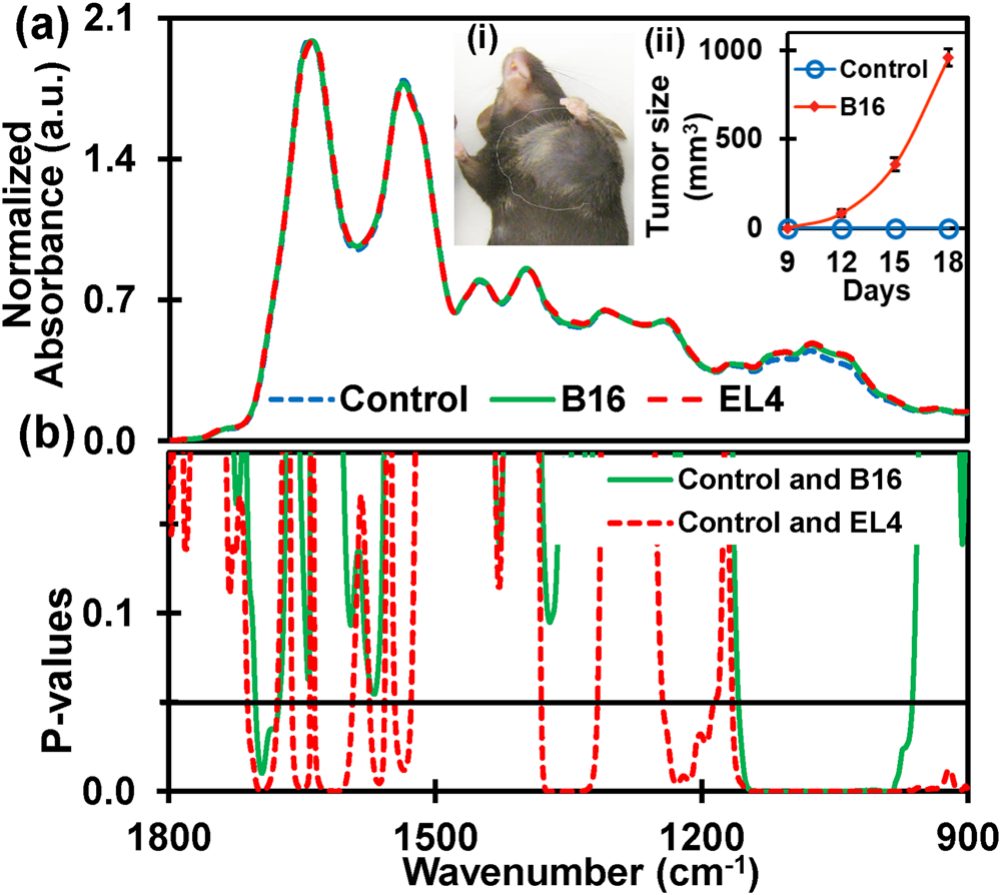
The discriminatory region of infrared absorbance spectra. (**a**) Average normalized ATR-FTIR spectra of serum samples extracted from EL4-lymphoma (n = 8), B16-melanoma (n = 8) mouse models in wild types and corresponding control types (n = 15). The inset (i) shows B16-melanoma mouse with tumor size approximately 1000 mm^3^ (day 18). Increase in volume of the tumor from the day 9 to day 18 of tumor inoculation in B16-melanoma mouse is as in inset (ii). Similar to the B16 mouse, elevation of tumor size is also monitored in EL4-lymphoma. The serum sample is extracted for both types of mice when tumor size becomes bigger than 1000 mm^3^. (**b**) Student’s t-test (two-tailed unequal variance) p-values of absorbance. Discriminatory region for lymphoma with higher significance (p < 0.05) are amide I of protein, amide II of protein, C-H bends of CH_3_/CH_2_ groups in α- and β- anomers, asymmetric phosphate I, and carbohydrates with predominant contributions nucleic acids (DNA/RNA via PO_2_^−^ stretches). Discriminatory regions of melanoma are amide I and carbohydrates with predominant contributions of nucleic acids.

Figure 1(a) shows the average normalized ATR-FTIR spectrum of air dried serum samples extracted from tumor-bearing mouse models of EL4 lymphoma (n = 8) and B16 melanoma (n = 8) in wild types and corresponding control types (n = 15). Using the student’s t-test, p-values (two-tailed unequal variance), the most discriminatory features of the spectrum within the spectral range 1800–900 cm^−1^, were extracted (Fig. 1(b)). Interestingly, the features observed for different groups enable the classification between control cases and malignant cases and between the two malignant cases of lymphoma and melanoma. Molecular assignments^26,28–32^ of five spectral bands showing discrimination of EL4 lymphoma from their control types, with higher significance (i.e. p-values < 0.05) are presented in Table 1. These are the bands originating from (i) amide I of protein, (ii) amide II of protein (iii) C-H deformation of CH_3_/CH_2_ groups, (iv) asymmetric phosphate I, and (v) Carbohydrates and nucleic acids. Similarly, two spectral bands showing the significant difference between B16 melanoma and their control types are also shown in the shaded regions of the table. Significant alteration in the amide I band and the complex band of carbohydrate and the nucleic acids are observed for B16 melanoma. The difference in the p-values observed between lymphoma and melanoma could be attributed to the difference in mechanism of each type of tumor development, while similarity could be attributed to common etiology^33^.

**Table 1:** Discriminatory infrared spectral bands of dried serum with biomolecular assignments (taken from references^26,28–32^).

Wavenumber region (cm^−1^)		Assignments
**i**	1700–1600	Amide I of proteins: (α-helical, β-pleated sheet, β-turns, random coils and side-chain structures), ν(C = O), ν(C-N), CNN.
**ii**	1480–1580	Amide II of proteins: (α-helical, β-pleated sheet, unordered conformation structures), δ(N-H), ν(C-N).
**iii**	1325–1380	C-H deformation: due to CH_3_/CH_2_ bending (groups in α and β anomers) of lipids and proteins.
**iv**	1190–1240	Asymmetric phosphate I: ν_as_(PO_2_^−^) of lipid phosphates.
**v**	1000–1140	Carbohydrates and nucleic acids: C-O, C-C stretch, C-H bend, deoxyribose/ribose DNA, RNA, ν_s_(PO_2_^−^).

### Protein secondary structures analysis by deconvolution of amide I band

**Figure 2:**
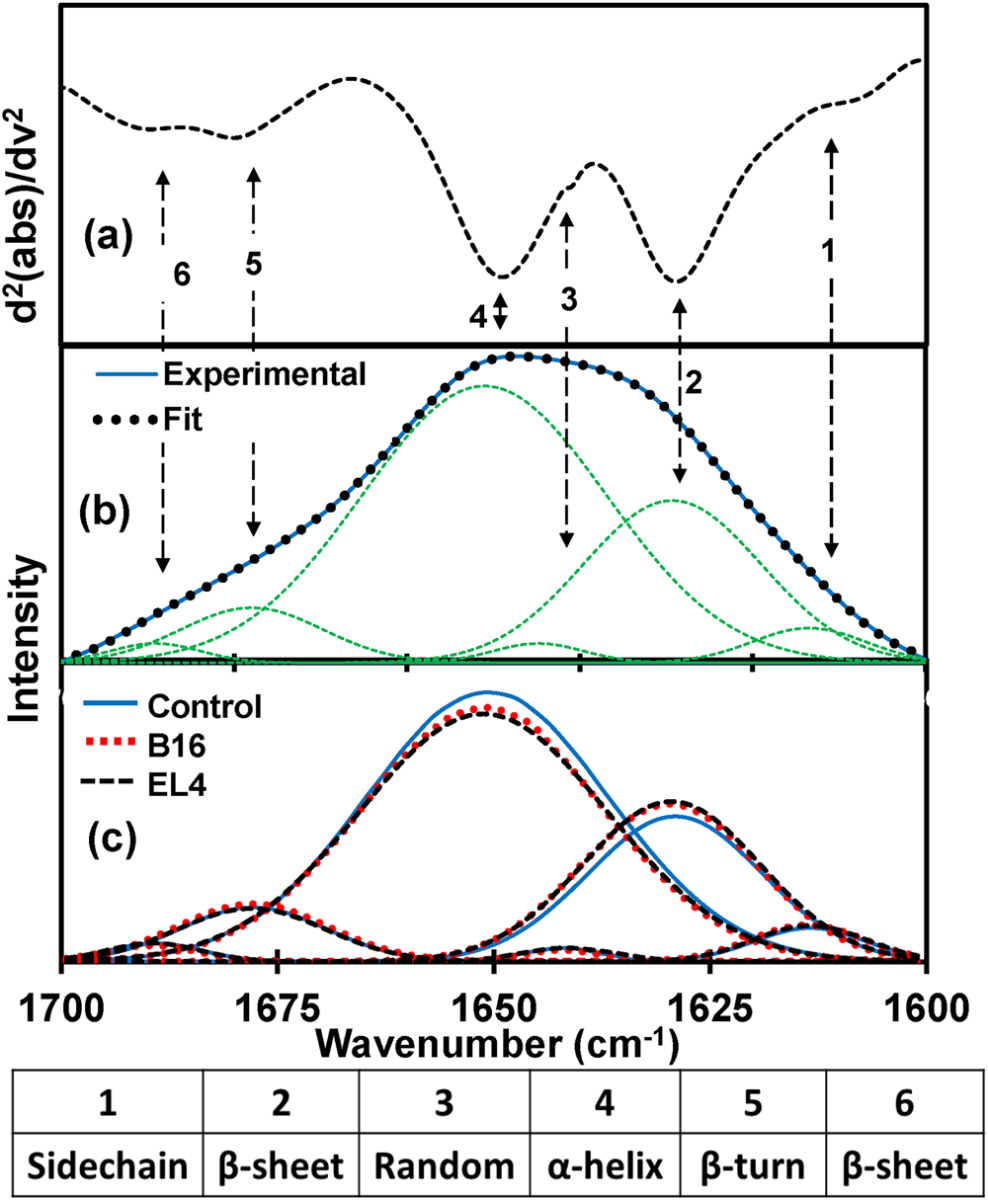
Protein secondary structure analysis. (**a**) The representative of the second derivatives of the ATR-FTIR absorbance spectra confined to amide I band. (**b**) Deconvolution of amide I region: baseline corrected spectra were fitted with six Gaussian band profiles by approximating number and position using the minima of second derivatives, which simulated fits to the experimental curve. Six Gaussian band profiles are assigned as (1) side chain (~1610 cm^−1^), (2) β sheet (~1630 cm^−1^), (3) random coil (~1645 cm^−1^), (4) α helix (~1652 cm^−1^), (5) β turn (~1682 cm^−1^) and (6) β anti-parallel sheet (~1690 cm^−1^) structures. (**c**) Averaged Gaussian function energy bands of each studied types which prove elevation of β sheet and drop off α helix structures due to malignancies, while other structures remain same.

Amide I band region with strong absorption is highly sensitive to the minor changes in molecular geometry and hydrogen bonding patterns of protein molecules. This sensitive vibrational band of protein backbone relates to protein secondary structural components and gives rise to different C = O stretching frequency for each structure^34^. Studies have shown that the secondary structure information obtained from the spectral deconvolution (or fitting)^34,35^ of the amide I band are in agreement with information from X-ray crystallographic structures of proteins^36–38^. Secondary structure analysis is done by the deconvolution of the experimental amide I band into component energy bands^39^. The minima of second derivatives of spectra (Fig. 2(a)) were used to approximate the position and number of Gaussian function energy profiles required to fit an experimental curve. Once the positions were determined, six Gaussian profile bands were used by minimizing Root Mean Square (RMS) error via a Levenberg-Marquardt function such that the simulated curve best fits the experimental curve as shown in Fig. 2(b). Energy bands at approximately 1652 and 1630 (in cm^−1^) have been assigned^40,41^ as vibrational modes of α–helix and β–sheet structural components respectively. The α–helix component integral value (area under the Gaussian band) decreases while the β–sheet component integral value increases simultaneously due to tumorigenesis (Fig. 2(c)). However, the integrals of component bands side-chain (~1610 cm^−1^), random coils (~1645 cm^−1^), β-turn (~1682 cm^−1^) and β–sheet with opposite alignments^40^ (1690 cm^−1^) do not show any appreciable change due to the tumor development.

**Figure 3:**
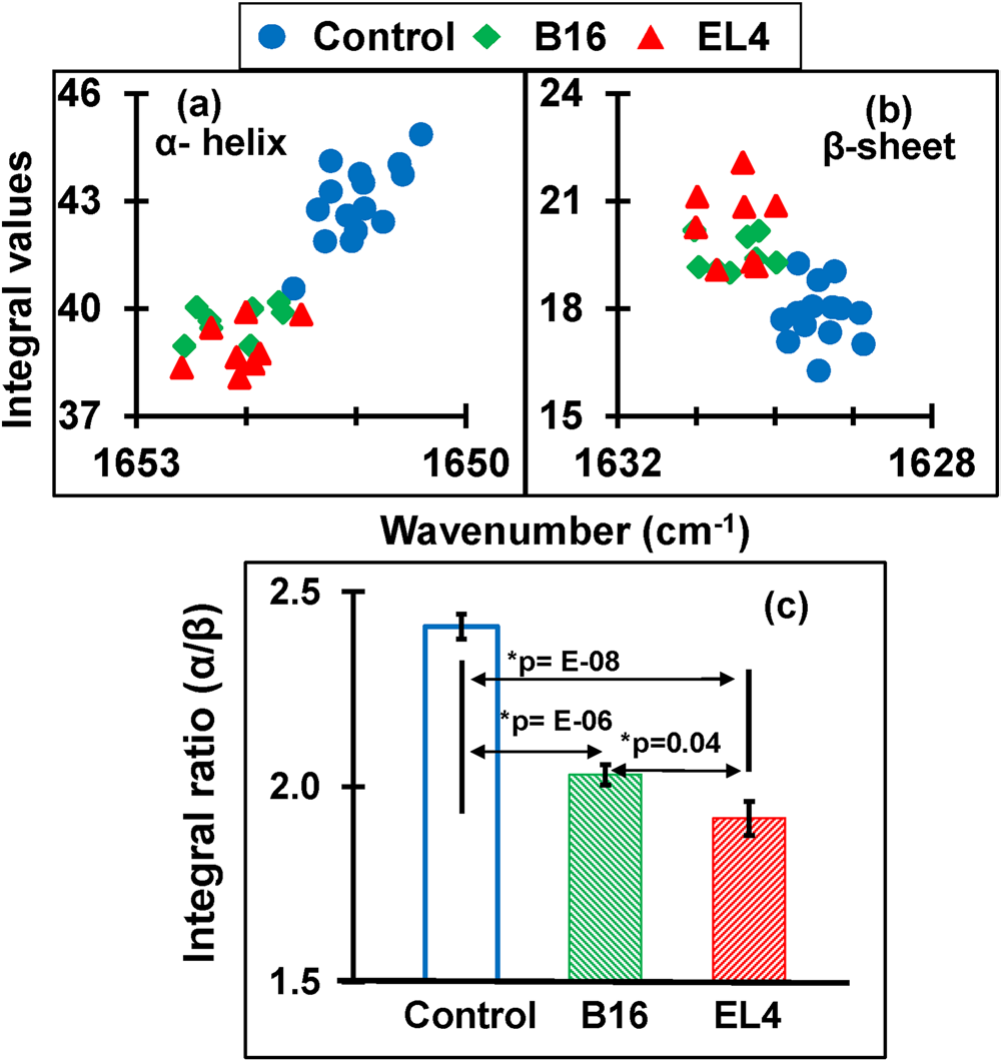
Plots of the protein secondary structures (α-helix, β-sheet) and their ratio. (**a**) Quantified integral (area covered) values of α-helix components are less for tumorous cases compared to control. (**b**) Integral values of β-sheet components are higher for tumorous cases compared to control (**c**) Bar graph representation of average integral ratios between α-helix and β-sheet for control, B16 and EL4. Significant alteration in integral ratio (α-helix/β-sheet) is found between control and tumorigenic case.

In order to demonstrate alterations in structural components due to malignancy, integral values of α–helical and β–sheet structures and their ratios were statistically analyzed. Figure 3(a) and (b) show the cluster plots of the integrals of α–helical and β–sheet structures respectively for the control, B16 and EL4 mice. These figures clearly demonstrate a separation between the corresponding integral values for the control and tumorous groups for β–sheet and α–helix. Furthermore, the ratio of integral values α–helix to β–sheet (Fig. 3(d)) is always less than the control values for both mouse models with greater than 99% significance.

### Amide I and II absorbance values

Amide I and Amide II are the two major bands of the infrared spectrum for protein interrogation in biological materials^28,29^. The intensity and position of these bands, determined by backbone confirmation of the hydrogen bonding pattern change with malignancies^13,42^. Amide I band position shifts towards the lower wavenumber due to malignancy (see supplementary materials Fig. S1). The average position of amide I representing control is at 1641 cm^−1^, B16 is 1640 cm^−1^ and that of EL4 is 1638 cm^−1^, but the position of amide II is exactly at 1538 cm^−1^ for all three types. Altered position of amide I is statistically significant for EL4 (*p = 0.001) while that of B16 is not significant (*p = 0.2). Similarly, altered ratio between amide I and amide II absorbance values is significant (*p = 0.01) for EL4 lymphoma but not (*p = 0.3) for B16 melanoma in comparison to the control groups.

### Nucleic acids and carbohydrate analysis

In the region 1140–1000 cm^−1^, there are plenty of overlapping vibrational modes of biological macromolecules^9^ with the major contribution of nucleic acids and carbohydrates^12^. Bands approximately at 1121 cm^−1^ arise from RNA absorbance, whereas the band at 1020 cm^−1^ arises from DNA absorbance^43^. The spectral band near 1080 cm^−1^ is due to ν_*s*_(PO_2_^−^), and the band approximately at 1056 cm^−1^ corresponding to the ν_*s*_(PO_2_^−^) absorbance of phosphodiesters of nucleic acids and the O-H stretching coupled with C-O bending of C-OH groups of carbohydrates^44^. Similarly, absorbance near 1033 cm^−1^ and 1076 cm^−1^ are due to the presence of glucose (C-O stretching carbohydrate, β-anomer) and mannose (C-O stretching carbohydrate α-anomer)^10^. Alteration in concentration of two sequences of basic genetic materials- (a) RNA (which play an active role in protein synthesis) and (b) DNA (which is primarily involved in the storage, copying and transferring genetic information), has been already reported from the tissue analysis of NHL^43^ and subcutaneous melanoma^45^. Due to the fluctuation in these biomolecules, there is a dissimilarity between malignant groups from their control types. In order to verify these dissimilarities, we have used Hierarchical Cluster Analysis (HCA) along with spectral deconvolution within this spectral range.

**Figure 4:**
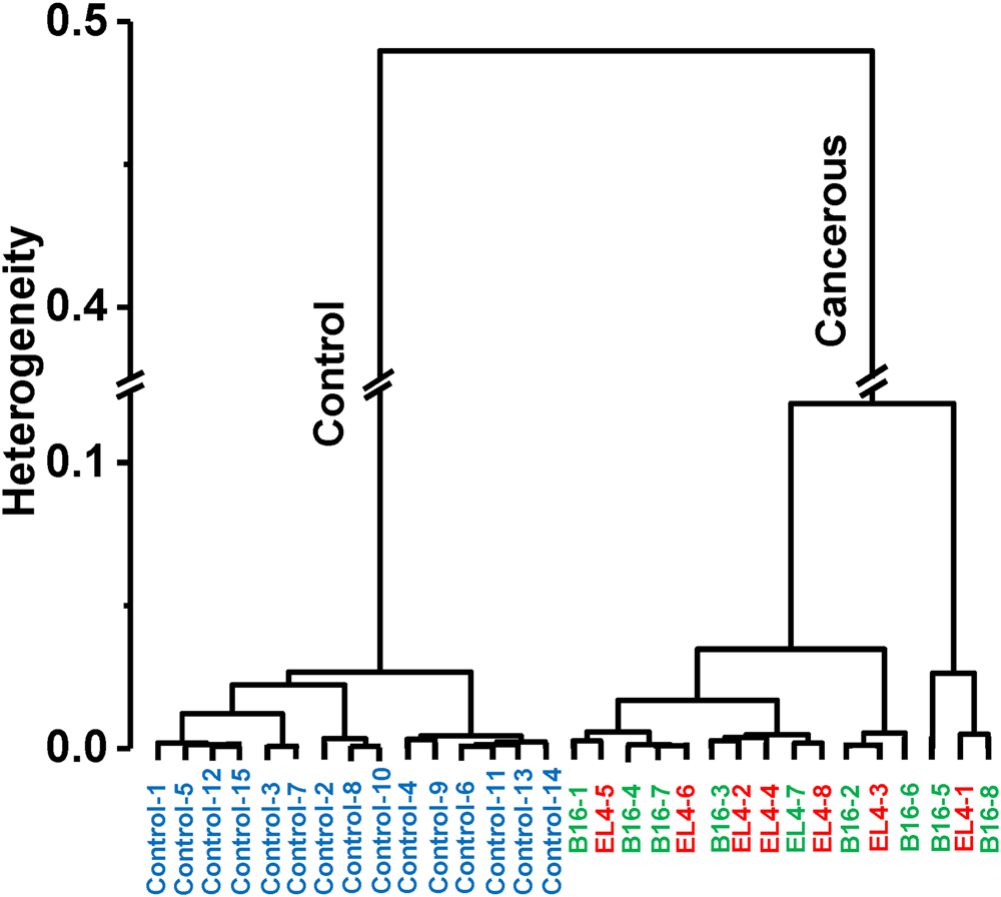
Dendrogram of hierarchical cluster analysis. Dendrogram tree diagram performed within spectral range 1140–1000 cm^−1^, by using Ward’s algorithm and squared Euclidian distance measurements. The spectra are correctly classified. Control spectra appear grouped together, which describes a high degree of similarity within the groups. Similarly, there is a distinct clustering in the cancer spectra in two subgroups showing the higher degree of heterogeneity between cancerous spectra.

HCA is commonly employed to identify the similarities between the FTIR spectra by using the distances between spectra and aggregation algorithms^14^. The dendrogram of HCA is performed with ATR-FTIR spectra of control, B16, and EL4 mice, are shown in Fig. 4. Dendrogram tree diagram performed using spectral region of nucleic acids and carbohydrates, 1140–1000 cm^−1^, using Ward’s algorithm and squared Euclidian distance measurements, allow us to visualize of overall grouping structure, including the sub-groups. The distinct cluster for the control spectra which are grouped together, describing a high degree of similarity within the groups. Similarly, there is a distinct clustering in the cancer spectra showing the higher degree of heterogeneity between spectra of cancerous groups.

**Figure 5:**
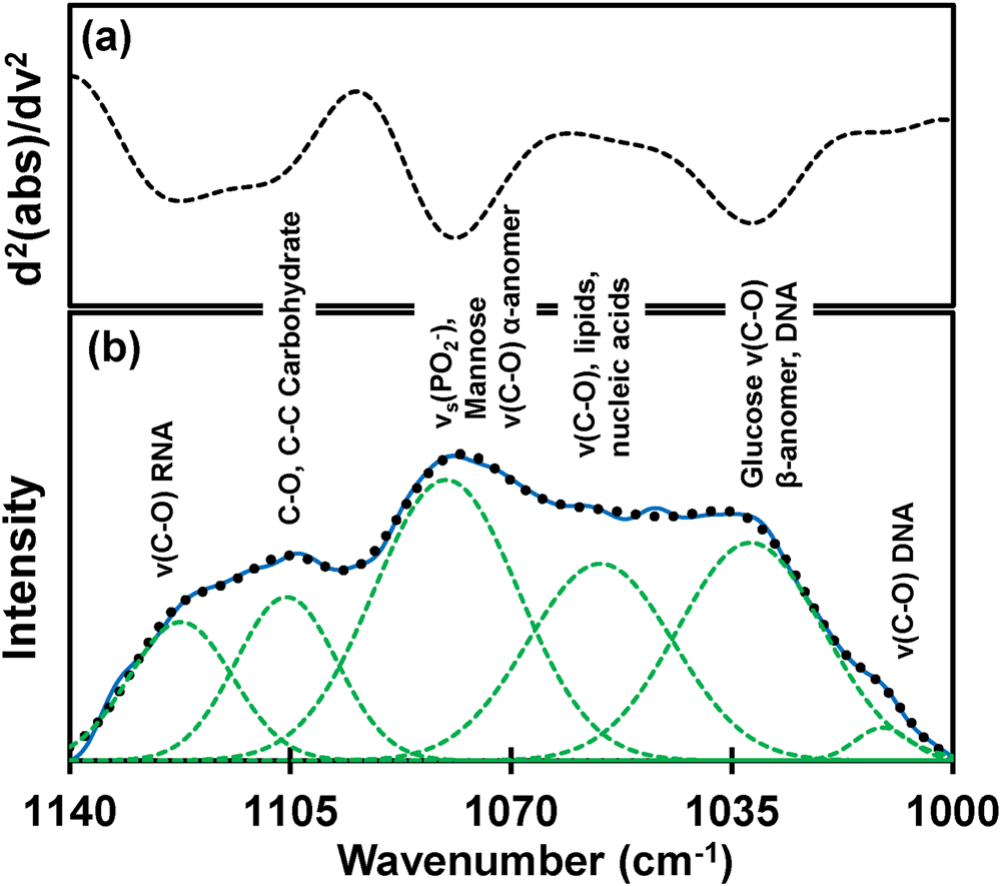
Analysis of carbohydrates and nucleic acids. (**a**) The representative of the second derivatives of the ATR-FTIR absorbance spectra confined to 1140–1000 cm^−1^. (**b**) Deconvolution of spectral range into six Gaussian band profiles by approximating number and position using minima of second derivatives.

**Figure 6:**
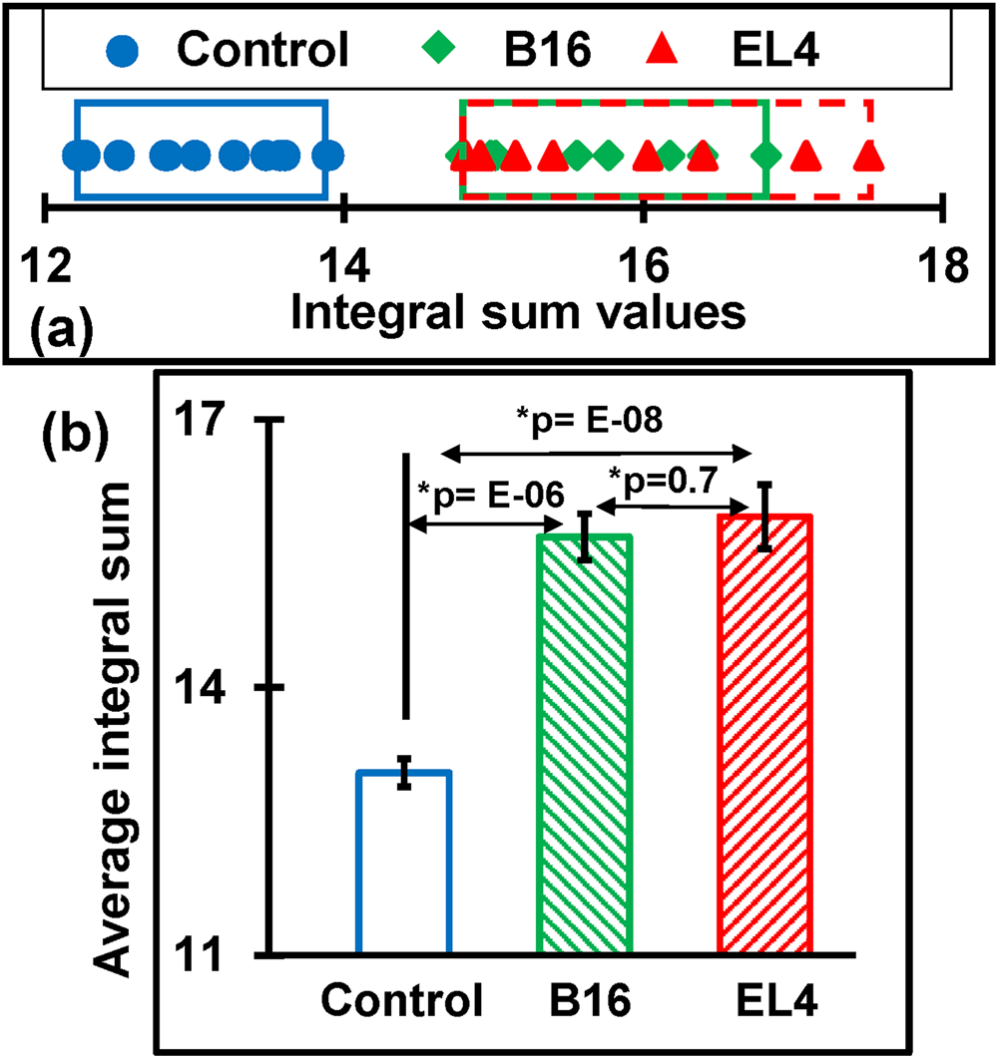
The integral sum of Gaussian energy profiles used to fit experimental curve within 1000–1140 cm^−1^. (**a**) The calibration curve obtained after adding integral values of energy profiles used to fit experimental curves. The sum of integral values of control groups cluster within the approximate range 12–14, B16 covers the range 15–17 and EL4 covers 15–18. (**b**) Bar graph representation of average value of integral sum which shows significant difference between control and tumorigenic case.

Furthermore, to quantify tumor-associated alteration within this complex spectral region of 1140–1000 cm^−1^, deconvolution of experimental spectra into Gaussian function band profiles is further employed. Six Gaussian function energy band profiles (Fig. 5(b)) are used to fit the spectra by approximating number and position using the minima of second derivatives (Fig. 5(a)). The sum of the integral areas covered by six bands (integral values) is then statistically analyzed to evaluate the tumor-associated alteration in the serum. A calibration curve is obtained, as shown in Fig. 6(a) between control and tumorous groups. A clear separation between control (12–14) and cases of tumorigenicity B16 (15–17) and EL4 (15–18) is found while adding the integral values. Bar graph representation of these values with significance greater than 99% is shown in Fig. 6(b).

## Discussion

The results of the present study show remarkable differences (Table 2) between the ATR-FTIR spectra of serum samples representing tumor-bearing mouse models of melanoma (n = 8) and NHL (n = 8) from their control (n = 15) types. The differentiating signatures between spectra are obtained by observing (i) p-values comparison, (ii) the spectral position and ratio analysis of amide peaks (iii) the fit of the experimental spectra and (iv) the employment of multivariate analysis (HCA). This difference between control and tumorous cases is evident through the gradual changes in the intensities of the absorption of mainly proteins, carbohydrates and nucleic acids in the serum. It is noted that serological tests show the alterations of certain proteins, peptides, and nucleic acids (DNA, mRNA) for patients with melanoma^46^ and lymphoma^47^. Manifestations of these alteration in biomolecules (serological markers) are most likely for the tumor-induced alteration in identifying spectral markers.

**Table 2:** Quantified values of discriminatory features.

Feature	Spectral deconvolution				Amide I and II		Absorbance values	
	Amide I 1600–1700 cm^−1^			Mixed region 1000–1140	Position amide I	Ratio: abs Amide I/II	ν_as_ (PO_2_^−^) ~1212 cm^−1^	C-H def. ~1335 cm^−1^
	Integral values		Ratio (α/β)	Integral sum				
	α-helix	β-sheet						
Control	40.6–44.9	16.3–19.3	2.2–2.7	12.2–13.9	1640–1645	1.10–1.13	0.42–0.44	0.54–0.59
B16	38.9–40.2	19–20.2	1.9–2.1	14.8–16.8	1638–1644	1.10–1.15	0.42–0.45	0.54–0.59
EL4	38.1–39.9	19.1–21.1	1.7–2.1	14.8–17.1	1636–1642	1.12–1.15	0.43–0.46	0.58–0.59

Clear separation can be seen between control and cancerous cases (both B16 and EL4) while comparing integral values of α-helix, β-sheet structure components, and their ratios. Similarly, altered position of amide I peak, amide I/amide II ratio and absorbance values at 1212 and 1335 cm^−1^ show significant difference only between EL4 and control.

Herein, this is an experimental demonstration of rapid and reliable spectroscopic technique for the discrimination of B16 melanoma and EL4 lymphoma mice from their control types. B16 murine tumor model remains an indispensable for metastasis and therapeutic studies of human melanoma skin cancer^48^. Similarly, development of EL4 murine tumor model considered as a huge benefit^49^ to the human NHL research cancer. This work is thus expected to lay a foundation for further research which could lead to the development of diagnostic techniques for future health care of cancer patients of melanoma and lymphoma using body fluid samples that can be collected with relatively low risks. It is thus critical to extend the present study to human patients for the assessment of disease status and personalized drug management. Furthermore, the study of temporal variation in spectral marker signatures is important for tumor grading, sub-typing and assessing the heterogeneity. Further work is in progress (i) to investigate temporal variation in serum components along with the progression of the disease by increasing sample size, (ii) identify the alteration in spectral markers using human patients, and (ii) to integrate data analyzing software into the narrow multiband detector. After setting a calibration curve of unique spectral markers for NHL or subcutaneous melanoma, bulky instrumentation will be avoided using specific multiband infrared detectors capable of simultaneous detection in the expected narrow bands. Recent advances in infrared technology allow the operation of multiband detectors at room temperature^50^. Complex statistical analysis of identifying spectral markers of NHL or melanoma can also be integrated into the clinical tool as a software application into the computer program. In terms of clinical application, we can anticipate that the potential technology can be further developed into a personalized diagnostic tool in which patient-to-patient and within a patient over time (due to health conditions or other factors) differences in molecular signatures would allow the assessment of disease status and personalized drug management. To be used as a patient to patient screening test, a normal range of spectral markers unique to the particular disease should be set by using a statistically significant set of normal serum samples. These average normal values can be incorporated into the program which can identify the deviations of the test sample from the average values. Technological advancement of ATR-FTIR spectroscopy of serum sample to discriminate normal and tumorous conditions will thus supports to increase compliance rate eligible population for tumor screening and to make physician decision for advanced histological examination using biopsy.

## Materials and Methods

### Mouse tumor models

C57BL/6 J mice (6–8 weeks, 20–22 g, the Jackson Laboratory) were engrafted with B16 melanoma or EL4 lymphoblast via subcutaneous (s. c.) route with 2 × 10^5^ of each cell line. B16 and EL4 cells were obtained from American Type Cultural Collection (ATCC) and maintained in DMEM with 10% FBS prior to use. Mice were euthanized after 3 weeks of tumor inoculation, when tumors were larger than 1000 mm^3^ in size (see Fig. 1, inset (i)). Serum samples from tumor-bearing mice and healthy mice were isolated and stored in −80 °C until analysis. All experiments using animals described in this study were approved (protocol number: A17015) by the Institutional Animal Care and Use Committee (IACUC) of Georgia State University, Atlanta, GA and experiments were conducted according to the guideline of Office of Laboratory Animal Welfare (OLAW), Assurance number: D16–00527(A3914-01).

### Fourier transform infrared spectroscopy

A Bruker Vertex 70 FTIR spectrometer series with KBr beam splitter and Deuterated Tri-Glycine Sulfate (DTGS) pyroelectric detector was used. The spectrometer was fixed with an MVP-Pro ATR accessory from Harrick-Scientific having diamond crystal (1 mm × 1.5 mm) as an internal reflection element and configured to have a single reflection of the infrared radiation. In all measurements, medium Blackman-Harris apodization function was employed with a resolution of 4 cm^−1^ with zero filling factor 4 to provide the best resolving ability with a minimum signal-to-noise ratio. Furthermore, for the optimization of the detector response and for the prevention of its saturation, aperture size is set to 2.5 mm.

### Sampling and scanning

ATR crystal was first cleaned using sterile phosphate buffered saline followed by ethanol. A cleanness test was then conducted, where the absorbance spectrum obtained without a sample to ensure it have no signal peaks higher than the environmental noise level. Background measurement was then performed prior to each spectral measurement by scanning a clean diamond crystal surface, and having its value subtracted from the sample signal spectrum. After setting these parameters, serum samples of one microliter volume were deposited on the crystal surface and allowed to air dry (~8 minutes) at room temperature. As the scanning runs, an evanescent wave with an approximate penetration depth of ~2.5 microns (for mid-IR) interacts with the sample. Each sample was scanned multiple times to get eight (or more) high-quality spectral curves, and the last six reads of the 100 co-added scans for each sample (total of 600 scans) were averaged.

### Spectral analysis

Using OPUS 7.2 spectroscopy software, all the spectra were internally normalized^12^ by scaling within the fingerprint region 1800–900 cm^−1^. In these normalized spectra, the absorbance values of amide I band position (~1642 cm^−1^) is 2 AU (corresponding to ~99% absorption) according to the Beer-Lambert algorithm. The significance of difference in absorbance values between control and diseased cases at different spectral marker positions were then tested by using the student’s t-test (two-tailed unequal variance) p-values. The significance test is then followed by the discrimination of protein secondary structures by deconvolution of the spectra into Gaussian function energy bands within the amide I band position 1700–1600 cm^−1^. Using OriginPro 2015 software, Hierarchical Cluster Analysis (HCA) was employed to identify the similarities between the spectra using the range of 1140–1000 cm^−1^. This spectral region has been studied before through the use of tissue biopsy while discriminating lymphoma^43^ and melanoma^45^ from control groups. Spectral deconvolution within the range was also completed to quantify spectral dissimilarity.

## Electronic supplementary material

Supplementary Information
