# Supplementary material for: ATR-FTIR spectral discrimination between normal and tumorous mouse models of lymphoma and melanoma from serum samples
Source: Sci Rep. 2017 Dec 5;7:16993. doi: 10.1038/s41598-017-17027-4 (PMC5717051; doi:10.1038/s41598-017-17027-4)
Supplement: Supplementary file 1 — Supplementary Information [file 41598_2017_17027_MOESM1_ESM.pdf]

Supplementary Information

ATR-FTIR spectral discrimination between normal and tumorous mouse models of lymphoma and melanoma from serum samples

Hemendra Ghimire[1], Mahathi Venkataramani[2,3], Zhen Bian[3], Yuan Liu[2,3] and A. G. Unil Perera[1,2,]*

*Corresponding Author: E-mail: uperera@gsu.edu, Phone: 4044136037, Fax: 4044136025

[1] Department of Physics and Astronomy, GSU, Atlanta, GA 30303, USA
[2] Center of Diagnostics and Therapeutics, Georgia State University, Atlanta, GA 30302, USA
[3] Center for Inflammation, Immunity and Infection, Georgia State University, Atlanta, GA, 30303, USA

Supplementary Results: Position and Ration Analysis of Amide Peaks (I and II)

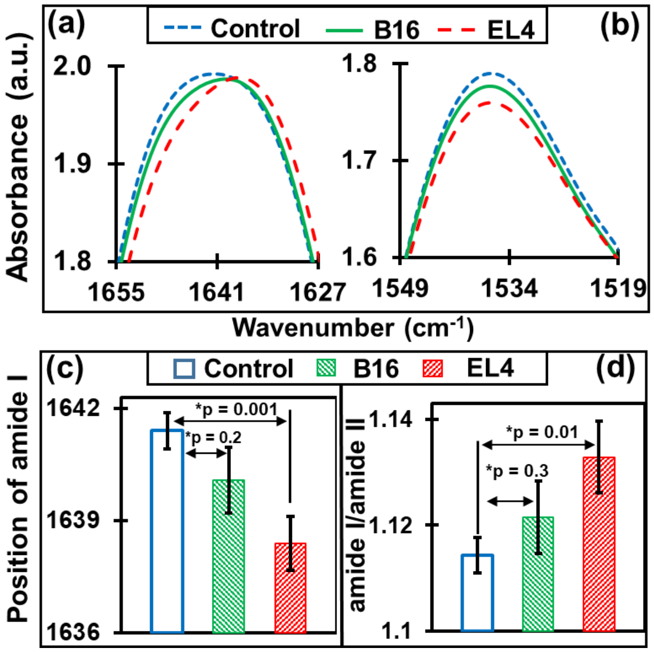

Figure S1. Position and ratio analysis between amide I and amide II. (a) Amide I peak shifts towards the lower wavenumber in malignant cases. The mean position of amide I for control is 1641 cm^{-1}, B16 is 1640 cm^{-1}, and EL4 is 1638 cm^{-1}. (b) Shows the amide II spectral band position and decreasing trend of absorbance values in cancerous cases. (c) Bar graph representation of the shift of the position of amide I group with higher significance for EL4 but not B16. (d) Alteration in the ratio (amide I/amide II) due to malignancy. Substantial difference is found between control and EL4 lymphoma, but difference is less for B16 case.
